# Tuberculosis of the eye, case series study

**DOI:** 10.25122/jml-2021-0343

**Published:** 2022-08

**Authors:** Zeena Adnan Abd

**Affiliations:** 1 Surgery Department, Ophthalmology Unit, School of Medicine, Al-Nahrain University, Baghdad, Iraq

**Keywords:** ocular tuberculosis, uveitis, anti-tuberculous drugs, choroiditis, ATT – Anti tuberculous treatment, BCG – Bacillus-Calmette-Guerin, ELISA – Enzyme linked immunosorbent assay, F – Female, IGRA – Interferon-gamma release assay, IOP – Intraocular pressure, M – Male, OCT – Optical coherence tomography, PCR – Polymerase chain reaction, TB – Tuberculosis, TST – Tuberculin skin test

## Abstract

Tuberculosis of the eye represents a challenge throughout the world, and there is a continuous debate about its pathophysiology, diagnosis, and treatment. The present research represents an interventional prospective study focusing on the variable clinical presentations, and the diagnostic and therapeutic characteristics of ocular tuberculosis. Fifteen eyes from nine cases were diagnosed with ocular tuberculosis, treated, and followed up between 2010 and 2020. The diagnosis was based on (1) a compatible clinical picture, (2) highly positive Tuberculin skin test or a positive IGRA test (Interferon-Gamma Release Assays), (3) a dramatic response to anti-tuberculous drugs without systemic steroid. Mean age was 41.22±13.64 years; eight cases were females 89.8%, one male 11.1%. Only one case had preexisting pulmonary tuberculosis. Bilateral ocular involvement occurred in two thirds of cases (66.7%). The most common clinical presentation was intermediate uveitis (33.3%), followed by multifocal choroiditis (20%). All cases were cured without relapse for the 2–10 years of follow-up, after taking oral anti-tuberculous drugs for 6–12 months. No systemic steroids were given, only topical steroid drops, as indicated. In conclusion, ocular tuberculosis is a mysterious condition with a wide-range of clinical presentations and should be considered in the differential diagnoses of any type of intraocular inflammation, or any unexplained reduction in vision. Oral anti-tuberculous drugs with or without topical steroids are sufficient to improve vision, produce, cure, and prevent relapse.

## INTRODUCTION

Tuberculosis (TB) has re-emerged as a global health problem. It is a chronic, slowly progressive, granulomatous infection caused by Mycobacterium tuberculosis. Tuberculous uveitis is a readily treatable disease but the consequences of delayed diagnosis can be devastating for the patient [1]. Mycobacterium tuberculosis is an acid-fast bacillus that usually affects the lungs, but it can also affect many other organs including the gastrointestinal, cardiovascular, musculoskeletal system, genitourinary tract, skin, central nervous system, and the eyes [2–3].

### Clinical manifestation

Ocular manifestations can be either due to direct infection with mycobacterium tuberculosis, or caused by indirect immune-mediated hypersensitivity reaction to mycobacterial antigens with no defined active systemic lesion [4–8]. Intraocular tuberculosis is a great mimicker of various types of uveitis. The clinical manifestations may include acute anterior uveitis, chronic granulomatous anterior uveitis with or without iris or angle nodules (granuloma), intermediate uveitis, vitrifies with macular edema, neuroretinitis, retinal vasculitis, solitary or multiple choroidal tubercles, monocular or binocular multifocal choroiditis, sub retinal abscess, endophthalmitis, and panophthalmitis [4–22].

### Diagnoses of ocular TB

It is difficult to make a definitive diagnosis of ocular TB. Therefore, the disease is frequently misdiagnosed and usually recognized after a significant delay [19]. The absence of clinical manifestation of pulmonary TB does not exclude the possibility of ocular TB, due to the fact that around 60% of patients with extrapulmonary TB have no signs of pulmonary TB [23]. In various studies, diagnoses of ocular TB were built on:

- A compatible clinical picture that does not fit any specific ocular or systemic disease;
- Strong positive Mantoux test (tuberculin skin test) more than or equal to 15 mm of indurations with or without necrosis;
- Clinical response to anti-tuberculous therapy (ATT) with no relapse [19–23].

### Tuberculin skin test (TST)

Tuberculin skin test dates back to the 19^th^ century, but it is widely used as an important step in diagnosing tuberculosis. However, its interpretation remains challenging and controversial, as it is affected by various factors including age, immunological status, coexisting illness, and many others [24]. Robert Koch was the first to describe the tuberculin reaction in 1890. Tuberculin is a glycerol extract of the tubercle bacilli. The precipitate is obtained from sterilized concentrated cultures [25]. False positive reactions can be seen in patients who have been infected with different species of mycobacteria [26, 27]. Moreover, false positives can be encountered in the cases of touching and scratching of the injected area, hypersensitivity, allergic reaction, or an error by the healthcare personnel [15]. False negative results can also be encountered [17].

### IGRA test (Interferon Gamma Release Assay)

IGRA (Interferon Gamma Release Assay) is a test executed *in vitro*, for cell-mediated immune response, to measure Interferon Gamma (IFN-γ) released from T cells after being stimulated by antigens specific to the mycobacterium tuberculosis (except BCG subs trains) [28]. Therefore, this test is not affected by BCG vaccination or by infection with nontuberculous mycobacteria, other than M. marianum, M. kansasii, M. szulgai, and M. flavescent. However, not all non-tuberculous mycobacteria have been studied for detecting cross-reactivity [29].

### Other diagnostic methods for ocular TB

#### Enzyme linked immunosorbent assay

One of the most abundant, most antigenic, and best studied cell wall component of tubercle bacilli is the purified cord factor, respectively trehalose-6,6'-dimycolate, against which antibodies can be detected, with utility in the serodiagnosis of pulmonary and ocular TB [8].

#### Polymerase chain reaction (PCR)

Due to the paucibacillary nature of ocular tuberculosis, there are many limits for detection of the tuberculous microorganisms using conventional techniques like smears and cultures. Therefore, polymerase chain reaction (PCR) may be a useful alternative for a definitive diagnosis of the disease [17]. Nonetheless, many challenging limitations do exist, including:

- Lack of adequate sample volume (aqueous or vitreous) with the associated additional morbidity [30];
- Non-uniform distribution of bacteria in these specimens [30];
- Presence of PCR inhibitors [30];
- Lack of the proper gold standards for the evaluation of the test [30];
- Previous attempts for PCR proven diagnosis of ocular TB were associated with low positive rates 33.3% to 66.6%, as initial PCR studies were based on single and not multiple targets [31, 32].

### Treatment of ocular tuberculosis

The standard treatment for ocular TB is the four-drug therapy (Isoniazid, Rifampicin, Ethambutol, and Pyrazinamide) given over a period of 6-9 months. Treatment failure was defined as the recurrence of the disease within six months of ATT completion. Cases with panuveitis, intermediate uveitis, and those on immunosuppressive (steroid) therapy had higher incidence of treatment failure [33]. Once a decision is made, the treatment should not be stopped due to lack of response after two months, unless another diagnosis is offered [34], and it should continue for at least 6–9 months [35]. Treatment failure has been difficult to define. Some proposed that failure is inability to decrease oral corticosteroids to a dose less than 10 mg/day, or to taper topical steroids to less than two times a day and recurrence or persistence of inflammation within the first 6 months after discontinuation of anti-tuberculous treatment [36].

### Objectives

The main objectives were to research in detail the variable clinical presentations of fifteen eyes from nine cases of ocular tuberculosis, the methods used for diagnosis, drugs used in treatment, and to analyze these data in order to help understand this rare, mysterious, but potentially blinding disease.

## Material and Methods

Our case series study included 15 eyes from nine patients with ocular tuberculosis, in which the diagnosis was made depending on:

- A compatible clinical picture: which is a suspicious clinical picture that does not fit any specific ocular or systemic disease;
- A highly positive TST (a minimum of 15 mm of induration, reaching up to 40 mm) or a positive IGRA test;
- A dramatic response to oral anti-tuberculous drugs within the first month of treatment and without giving systemic steroids.

All patients underwent complete ocular examination including testing of visual acuity by the standard Snellen chart, IOP (Intraocular pressure measurement), slit lamp for anterior, and posterior segment with dilated fundus examination. Some cases were sent for retinal imaging by OCT (optical coherence tomography) and fundus imaging for documentation and follow-up. Relevant blood investigations were done according to the clinical presentation. The patients were then sent to a tuberculosis center for performing TST, the IGRA test, and checking for the presence or absence of pulmonary TB by physical examination, chest X-ray and sputum for acid fast bacilli, when indicated. When the suggestive clinical picture was accompanied by a highly positive TST result (that is equal to or more than 15 mm of induration), then the decision was to start oral four drug ATT (Isoniazid, Ethambutol, Rifampicin, and Pyrazinamide), after performing the baseline investigations. The patients were reviewed at the eye clinic after one month to monitor the clinical response. Those with dramatic clinical response in terms of signs and symptoms were kept on treatment for 6 to 9 months. The duration of the treatment was individualized according to the patient's clinical follow-up. For the first 3 months of treatment follow-up was carried out monthly, and then became two times per month. Furthermore, complete ophthalmological examination was carried out every 3–6 months looking for signs of recurrence. Follow-up period ranged from 2 to 10 years.

## Results

**Figure 1:**
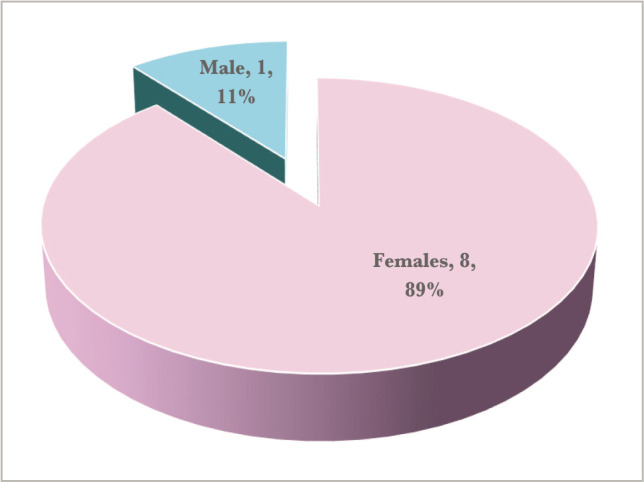
Sex distribution.

**Figure 2:**
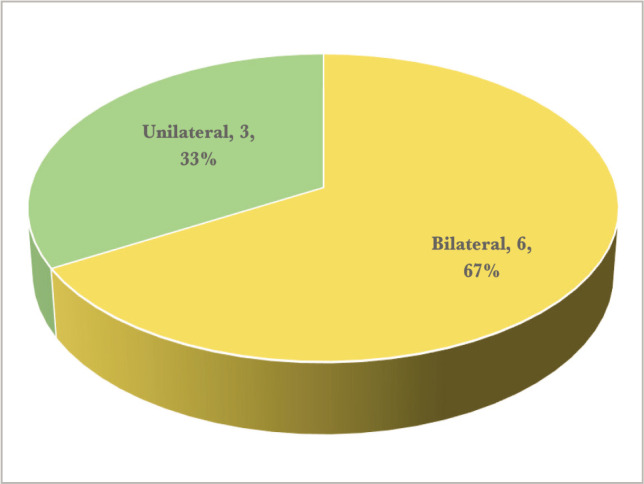
Unilateral *versus* bilateral eye involvement.

**Figure 3:**
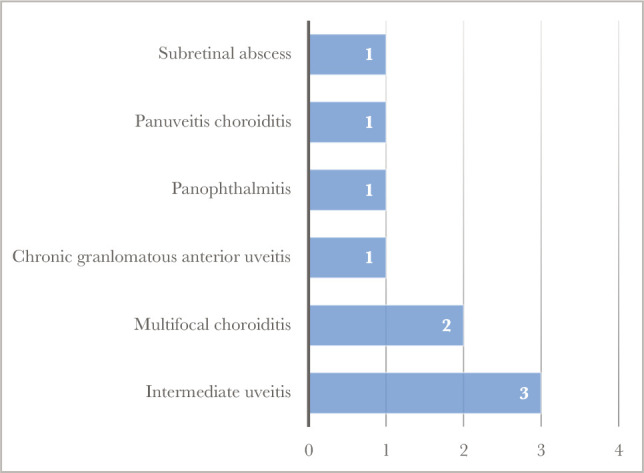
Number of cases in relation to the type of clinical presentation.

Table 1 highlights the mean age for the patients, which was 41.2 years, while Figure 1 showcases the gender distribution, where all but one case were female. Furthermore, in about two thirds of our cases, eye involvement was bilateral, as shown in Figure 2. There were highly variable clinical presentations, as there were six different types of ocular involvement. The distribution of cases according to the type of clinical presentation in the nine cases included in our study is shown in Figure 3. Only one case had positive pulmonary TB, as revealed in Table 2.

**Table 1:** Age distribution.

	Median	Mean±SD	Minimum	Maximum
**Age (yr)**	41.0	41.22±13.64	16.0	60.0

**Table 2:** Sex and age in relation to laterality, mode of presentation and presence or absence of pulmonary TB.

Sex	Age	Side	Pathology	Pulmonary TB
**F**	54	Bilateral	Chronic granulomatous anterior uveitis	Negative
**F**	33	Bilateral	Intermediate uveitis	Negative
**F**	53	Bilateral	Intermediate uveitis	Negative
**M**	33	Bilateral	Multifocal choroiditis	Negative
**F**	60	Bilateral	Pan-uveitis with choroiditis	Negative
**F**	41	Bilateral	Sub-retinal abscess	Negative
**F**	35	Unilateral	Intermediate uveitis	Negative
**F**	46	Unilateral	Multifocal choroiditis	Negative
**F**	16	Unilateral	Panophthalmitis	Positive

## Discussion

Many studies on ocular tuberculosis were implemented worldwide, especially in endemic areas, as it represents a devastating disease that can potentially lead to blindness in the absence of early detection and proper management. A multinational review from 2019 [37], including 251 patients with tuberculous retinal vasculitis found the mean age of patients to be 38.9 years, similar to that revealed in our research (41 years). However, in the collaborative study the cases were predominantly males (66%), compared to only 11% in the present research. This finding may present bias due to the small sample size and the research being conducted in one country. Furthermore, another source of bias can be represented by the singular pathology included in the collaborative study, namely retinal vasculitis. When considering response to treatment, eight out of the nine cases included in the present study showed dramatic response to ATT alone (88.8%) within the first month of treatment. The ATT was achieved without giving any systemic steroids. The treatment continued for 6 to 9 months according to individual factors (clinical response, laterality, severity of involvement). The efficacy and safety of ATT was analyzed in a meta-analytic research from 2016 [38], which reviewed 28 studies, including a total of 1917 patients with ocular tuberculosis. The research revealed a successful outcome for 85% of those receiving ATT alone, in comparison to a successful outcome of 82% for those receiving ATT and steroid medication concomitantly. In our study, non-recurrence was confirmed in all cases at 2–10 years follow-up. When discussing modes of presentation, ocular TB remains the big artist, and it should be considered in the differential diagnosis of any type of intraocular inflammation [39]. In our case series study, the most common clinical presentation was intermediate uveitis in three out of nine cases (5 out of 15 eyes), as two cases had bilateral involvement and one case was unilateral. The second most common clinical presentation was multifocal choroiditis, revealed in two cases (one bilateral and one unilateral). Other pathologies, each represented by one case, included sub retinal abscess, panuveitis with peripheral choroiditis, panophthalmitis, and chronic granulomatous anterior uveitis. In a case series conducted in 2001 [7], five different presentations were shown in the five cases presented, namely panophthalmitis, posterior uveitis with choroidal tubercular's, endophthalmitis, keratitis, and lid mass. In another case series study conducted between 2006 and 2015 [40], where patients had a mean age of 40.7 years, uveitis was the most common clinical presentation. There is poor correlation between pulmonary and ocular tuberculosis, among our nine cases, as only one patient had pulmonary tuberculosis. Similarly, in a study conducted in 2013 [41], seven out of 103 cases diagnosed with pulmonary TB showed signs of ocular involvement, and the most common ocular lesions were non granulomatous anterior uveitis and choroidal tubercles.

## Conclusion

Ocular tuberculosis has widely diverse clinical presentations. Diagnosis needs a high index of suspicion, so this affection should be kept in mind as a differential diagnosis in any type of ocular inflammation or even any unexplained reduction in vision. Highly positive TST or a positive IGRA test together with a compatible clinical picture is an indication to start anti-TB medication. If there is a noticeable clinical response, then treatment should continue for at least 6 months or more according to the follow-up. We do not recommend using oral corticosteroids from the start without documenting the clinical response to anti-TB drugs alone, as this would confuse the clinical picture, in the sense that it may mask or worsen different types of ocular infections and may make the justification for long-term anti-TB drugs difficult or uncertain. Nevertheless, controlled clinical trials are needed for true comparison. Therefore, our study supports the theory that presumed ocular tuberculosis results from true infection and proliferation with mycobacterial tuberculosis (inside or outside the eye). The use of topical corticosteroid drops may relieve symptoms in patients with uveitis, but it may worsen cases of tubercular scleritis resulting in panophthalmitis and probably perforation or phthisis. There is high-incidence of bilaterality, although involvement of the second eye may take months to years, and the earlier the diagnosis the better the outcome.

## Personal thanks

I would like to thank all the patients and their families for their great cooperation during the study period and their commitment for the many years of follow up. Also great thanks to medical teaching hospitals: Ibn-Alhaitham teaching eye hospital, Medical City and Al-Imamain Al-Kadhumain Medical City for being the places where the examination, management, and follow-up of patients was undertaken.
